# Antimicrobial use in hospitalized patients: a multicentre point prevalence survey across seven hospitals in Ghana

**DOI:** 10.1093/jacamr/dlab087

**Published:** 2021-07-12

**Authors:** Appiah-Korang Labi, Noah Obeng-Nkrumah, Nicholas T K D Dayie, Beverly Egyir, Eric Sampane-Donkor, Mercy Jemima Newman, Japheth Awuletey Opintan

**Affiliations:** 1 Department of Medical Microbiology, University of Ghana Medical School, College of Health Sciences, University of Ghana, Accra, Ghana; 2 Department of Medical Laboratory Sciences, School of Biomedical and Allied Health Sciences, College of Health Sciences, University of Ghana, Accra, Ghana; 3 Department of Bacteriology, Noguchi Memorial Institute for Medical Research, College of Health Sciences, University of Ghana, Accra, Ghana

## Abstract

**Background:**

Antimicrobial resistance (AMR) is a public health crisis of global proportions. Data is required to understand the local drivers of antimicrobial resistance and support decision-making processes including implementation of appropriate antimicrobial stewardship strategies.

**Objectives:**

To measure antimicrobial usage in hospitals in Ghana.

**Methods:**

Using the Global Point Prevalence instruments and processes, we conducted point prevalence surveys across AMR surveillance sentinel hospitals in Ghana, between September and December 2019. Hospital records of all inpatients on admission at 0800 hours on a specific day were reviewed for antimicrobial use at the time of the survey. Data on antibiotic use, including indication for use and quality of prescribing were recorded.

**Results:**

Overall prevalence of antibiotic use across the sentinel sites was 54.9% (*n = *1591/2897), ranging between 48.4% (*n = *266/550) and 67.2% (*n = *82/122). The highest prevalence of antibiotic use 89.3% (*n = *25/28) was observed in adult ICUs. The average number of antibiotics prescribed per patient was 1.7 (*n = *1562/2620), with the majority (66%, *n = *728/2620) administered via the parenteral route. The five most-commonly used antibiotics were metronidazole (20.6%, *n = *541/2620), cefuroxime (12.9%, *n = *338/2620), ceftriaxone (11.8%, *n = *310/2620), amoxicillin/clavulanic acid (8.8%, *n = *231/2620) and ciprofloxacin (7.8%, *n = *204/2620). The majority (52.2%; *n = *1367/2620) of antibiotics were prescribed to treat an infection, whilst surgical prophylaxis accounted for 26.1% (*n = *684/2620).

**Conclusions:**

We observed a high use of antibiotics including metronidazole and cephalosporins at the participating hospitals. Most antibiotics were empirically prescribed, with low use of microbiological cultures. High usage of third-generation cephalosporins especially for community-acquired infections offers an opportunity for antibiotic stewardship interventions.

## Introduction

Antimicrobial resistance (AMR) is on the increase worldwide.^1^ It is estimated that the overall short- and long-term impact of AMR is likely to be higher in low and middle-income countries (LMICs), especially those in sub-Saharan Africa,^2^ mainly owing to lack of therapeutic options.^2^ Major drivers of AMR worldwide include inappropriate antibiotic use in human and animal health and poor infection prevention and control practices. As part of global efforts to control antibiotic use and resistance, the WHO in 2016 launched the global action plan for the control of AMR^3^ with a call for member countries to develop their national AMR policy and action plans. Two major objectives of this global action plan are to strengthen knowledge through surveillance and research, as well as optimize the use of antimicrobial agents.^3^

Hitherto, different studies have highlighted high levels of AMR^4–6^ and antimicrobial use in Ghanaian hospitals,^7–9^ but no routine surveillance systems exist in the country. Subsequently, the Fleming Fund — an initiative from the United Kingdom Department of Health — is supporting the establishment of a national AMR surveillance programme as part of efforts to implement the Ghana national action plan on AMR.^10^ The surveillance programme is initially being implemented at seven hospital sites across the country. To complement the surveillance activities and for a better understanding of the drivers of AMR, we conducted a point prevalence survey (PPS) of antimicrobial use at hospitals participating in Ghana AMR surveillance. PPS is a standard WHO methodology that collects information on antimicrobial prescribing including indication for use, culture-confirmed diagnosis, and adherence to treatment guidelines.^11^ It allows for data collection at specific times but with standard procedures that permit data comparison across hospital sites, regions, and countries. PPS is particularly useful for the majority of LMICs, including Ghana, where medical records are largely paper-based and routine monitoring of antibiotic prescribing is a challenge due to the high workload as well as resource challenges confronting regular data collection. In this article, we report on the multicentre PPS that was done across all hospitals involved in the Fleming Fund Ghana AMR surveillance with a focus on the overall prevalence of antimicrobial use, types of antimicrobials commonly used, indication for use and quality of antimicrobial prescribing indicators.

## Methods

### Setting

**Figure 1.**
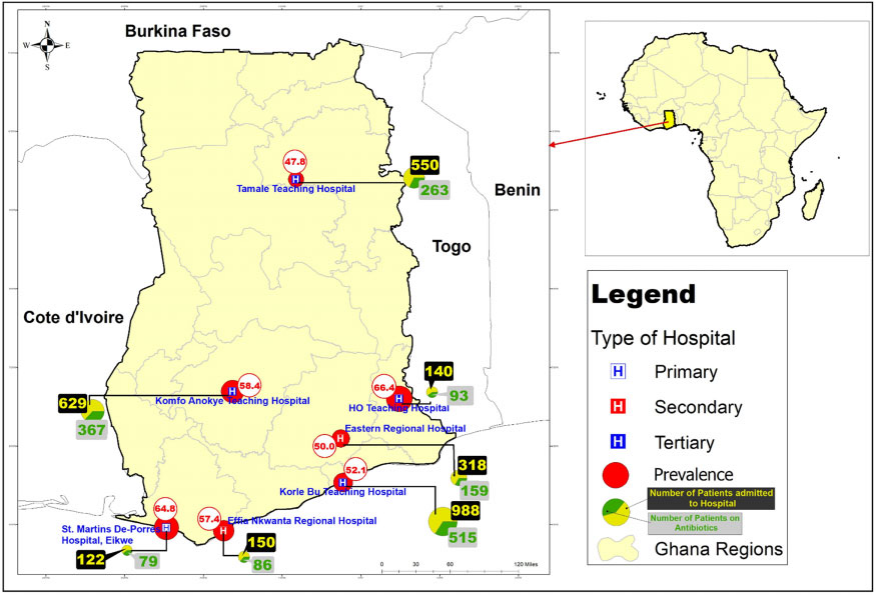
Prevalence of antibiotic use across study hospitals categorized as primary, secondary and tertiary. The diameter of the prevalence circle is proportional to the antibiotic use burden. Please note that St. Martins De-Porres Hospital is referred to as Eikwe District Hospital.

Figure 1 shows the locations of the seven hospital sites involved in the PPS and their level of healthcare delivery. Ghana, in general, has three levels of healthcare delivery. Primary care services refer to the work of general physicians who act as the first point of consultation. These include district, rural, community, and general hospitals. Secondary-level hospitals, often referred to as regional hospitals, are more differentiated and provide specialist medical care through referrals from primary healthcare professionals. Tertiary-level hospitals, mostly teaching hospitals, provide specialized consultive medical care through a referral from primary or secondary health professionals and perform most of the complex medical procedures. The hospitals in this study included: four tertiary care hospitals [the 2000 bed Korle-bu Teaching Hospital and the 350 bed Ho Teaching Hospital (both located in southern Ghana), the 1000 bed Komfo Anokye Teaching Hospital (in the middle belt of Ghana) and the 800 bed Tamale Teaching Hospital (located at the northern part of Ghana); two secondary care hospitals, the 420 bed Efia Nkwanta Regional Hospital and the 430 bed Eastern Regional Hospital (both located in southern Ghana); and a primary care hospital, the 200 bed Eikwe District Hospital (located in southern Ghana)]. All seven hospitals offer microbiology services including bacterial culture and susceptibility testing. None of the hospitals had an active antibiotic stewardship programme.

### Study design

This was a multicentre observational study. We reviewed the hospital records of all patients on on a ward at 8 o’clock in the morning of the survey according to Global PPS definitions (http://www.global-pps.com/documents/). All departments and units in the participating hospitals were included in the survey. The survey was conducted between September and December 2019.

### Study instruments

We used standardized instruments from the Global PPS platform for all data collection activities (http://www.global-pps.com/documents/). The Global PPS protocol aligns with the WHO PPS recommendations, is flexible and is ideal for use in LMICs, including Ghana. The Global PPS platform includes an optional freely available internet-based application with form-based user interfaces for data entry. The application checks erroneous data entry, such as double-entry of the same drug. The application also has built-in error and warning checks for data validation as well as real-time analysis tools for feedback and reporting. The Global PPS has worldwide coverage, and the online application permits for direct comparison of antimicrobial use patterns across hospitals from different regions.

### Study personnel and training

Pre-survey, a team of health personnel was gathered comprising the research investigators of this study and other healthcare professionals recruited from the participating hospital. The team included medical doctors, pharmacists, nurses, and laboratory personnel. We conducted a 1 day information and training session for staff participating in the PPS at each participating hospital. The Global PPS helpdesk, which hosts answers to frequently asked questions, was used to support the training sessions. Training included PPS terms and definitions, survey operations and data collection procedures. This was necessary to improve the reliability of study ﬁndings. The training was designed to introduce survey personnel to the objectives of the PPS, the purpose of each item on the data collection tool, such as the definition of terms, methods for assessment of individual patient data, and the roles and responsibilities of individual survey personnel. The training sessions were concluded with a pilot PPS of selected hospital wards on the day before the inception of the hospital-wide survey to allow for corrective actions.

### Data collection

The Global PPS protocol recommends that 100% of usable inpatient records be used. We, therefore, surveyed all patients in the hospital on the survey days. In-patients of any age on admission for more than 24 h were eligible for inclusion in the study, whilst patients attending daycare or with a stay of less than 24 h of ward admission were excluded. Medical records of patients admitted to the ward on or before 8 am on the day of the survey at a given hospital were examined within 12 h for current systemic antimicrobial use. Data on topically administered antimicrobials were not collected. All wards in a single hospital were surveyed once. The total timeframe for data collection in all wards within a hospital did not exceed 4 days. Patient and ward data were recorded on paper forms. Patient data were collected through a review of patient clinical notes and charts (electronic and paper). For each patient on at least one antimicrobial treatment, the patient-level data included age, gender, patients’ antimicrobial usage and reasons for use, dosage, dosing, route of administration, presence of active community- or healthcare-associated infections, results of routine microbiology tests performed, and the quality of antimicrobial prescribing. Ward data collected included the type of ward, the total number of beds and the number of patients admitted to each ward at the time of the survey.

### General terminologies

We classified all drugs administered to treat or prevent infection as anti-infectives. Two major categories were used: antibiotics and ‘other antimicrobials’. The former refers to conventional antibacterial agents for systemic use. The latter include antimycotics and antifungals for systemic use, drugs to treat tuberculosis, antimalarials, and antivirals. A prescription was defined as the use of one antimicrobial by one route of administration. Antibiotic agents were analysed using the WHO Anatomical Therapeutic Chemotherapy (ATC) 5th level classifications.^12^^,^^13^ Antibiotic prescriptions were categorized as either for treatment of infections or prophylaxis. The former included therapeutic antibiotics prescribed for community-acquired or healthcare-associated infections (respectively, infections with the onset of symptoms 48 h before or after hospital admission). Antibiotics administered for prophylaxis were determined as being for medical or surgical indications. When surgical prophylaxis was given, the duration of prophylaxis was recorded as either one dose or multiple doses given in 1 day or more than 1 day. The following age classification was used in the analysis: neonate ≤28 days, infants >28 days to <365 days, and paediatrics >1 to <14 years. Given that children aged >13 years in our settings are admitted to adult wards, we categorized patients aged >13 to <18 years as teenagers, >18 to ≤65 years as adults, and those older than 65 years as the elderly. Based on the global PPS protocol, we categorized hospital wards into six specialities: medical, surgical and ICUs for adults, neonates and paediatric patient populations. We also collected data to determine indicators for quality of antimicrobial prescribing. These included the use of C-reactive protein, procalcitonin, white blood cell counts, or any other biomarkers to support prescribing decisions. We also recorded documentation of the reason to start treatment in the patient’s notes and documentation of a stop or review date in the notes, and availability of local guidelines to advise on antimicrobial treatment. We documented antibiotic prescriptions as targeted if they were based on laboratory results for bacteria culture and susceptibility testing. Additional protocol and definitions used in the data collection can be found at http://www.global-pps.com/documents/.

### Data analysis and statistics

Data collected were entered onto the Global PPS platform but the data remained the property of the hospital. All captured data were anonymized within the database and safeguarded at the University of Antwerp (Antwerp, Belgium). For analysis, we included hospital-, ward-, and patient-level data from all seven participating hospitals. No data was excluded from any hospital. Data was exported from the online platform into an Excel database and subsequently imported into Statistical Package for the Social Sciences (SPSS version 21) for analysis. Categorical data were reported as the frequency with percentage while continuous data were presented as mean (with standard deviation) or median. Prescribed antibiotics were reported as the number of patients receiving at least one antibiotic per diagnosis. Prevalence of antibiotic use was defined as the number of patients receiving at least one antibiotic divided by the total number of patients on admission at the time of the survey. The patient to antimicrobial prescription ratio was calculated as the total number of patients surveyed divided by the total number of antimicrobial prescriptions.

Drug utilization 100% (DU100%) referred to the number of antimicrobials accounting for 100% of drug use. We ranked the drugs by volume of defined daily doses and determined how many antimicrobials accounted for the DU100% segment.

### Ethics

The study received approval from the Ethical Review Committees of the Ghana Health Service (GHS-ERC010/05/19), Komfo Anokye Teaching Hospital (CHRPE/AP/523/19), Korle-Bu Teaching Hospital (KBTH/MD/G3/19), and Tamale Teaching Hospital (TTH/R&D/SR/19/129). Informed consent was waived for the conduct of the study.

## Results

A total of 4387 patient beds were counted on the wards during the survey [primary care hospital, 199 (4.5%); secondary care hospitals, 709 (16.2%) and tertiary care hospitals, 3479 (79.3%)]. The total number of beds ranged from 199 to 1403 with a median bed size of 417 beds (IQR 392–1201). Over the study period, 2897 individual folders and charts of patients admitted to 55 wards were reviewed for the current use of antimicrobials. Of these, 122 (4.2%) patients were from a primary care hospital, 468 (16.2%) from secondary care hospitals, and 2307 (79.6%) from tertiary care hospitals.

### Prevalence of antibiotic use

Of the 2897 patients on admission, there were 2875 antimicrobials prescribed for 1591 patients (patient to antimicrobial prescription ratio, 1 : 1.8) (Table 1). The majority of the antimicrobials used were antibiotics (91.1%, *n = *2620/2875). Overall, 1562/2897 (53.9%) patients on admission received at least one antibiotic for systemic use on the day of the survey. Out of 1562 patients, 39.5% (*n = *617) received one antibiotic, the majority (53.7%, *n = *839/1562) received two antibiotics, 6.3% (*n = *99) received three antibiotics, and 0.4% (*n = *7) received four antibiotics. The average number of antibiotics per patient was 1 : 1.7 (*n = *2620 antibiotics/1562 patients). The median age of patients on antibiotics was 33 years. This varied from 1 day to 104 years (IQR: 22–50 years). The commonest route of antibiotic administration was the parenteral route (66%, *n = *1728/2620). Prevalence of antibiotic use varied between sites (from 47.8% in Tamale Teaching Hospital to 66.4% in Ho Teaching Hospital, Figure 1). Tertiary hospitals and secondary hospitals had 53.7% (*n = *1238/2307) and 52.4% (*n = *245/468) prevalence of antibiotic use, respectively (Table 2). In addition, the use of antibiotics varied by speciality (from 45.8% in paediatric intensive care to 89.3% in adult intensive care).

**Table 1.** Summary of antimicrobial prescriptions for 2897 patients on admission

Prescriptions	Number of patients (%)	Number of prescriptions (%)
Anti-infective agents	1591 (54.9)	2875 (100)
Antibiotics only	1439 (49.6)	2399 (83.4)
Antibiotics with ‘other antimicrobials’	123 (4.2)	221 (7.7)
At least one antibiotic drug^a^	1562 (53.9)	2620 (91.1)
Only ‘other antimicrobials’^b^	29 (1.0)	34 (1.2)
At least one ‘other antimicrobial’ drug^c^	152 (5.2)^d^	255 (8.9)
Anti-TB^e^	36 (1.2)	85 (2.9)
Antimalarials	66 (2.2)	70 (2.4)
Antivirals	34 (1.2)	67 (2.3)
Antifungals	30 (1.0)	33 (1.1)

a Prescribed antibiotics with or without ‘other antimicrobial drugs’.

b Given ‘other antimicrobials’ without any antibiotic prescription.

c Patients prescribed ‘other antimicrobials’ with or without antibiotics.

d The numbers of patients on anti-TB, antimalarials, antivirals, antifungals do not add up to 152 due to overlap in prescriptions.

e Anti-TB, antituberculosis drugs.

**Table 2.** Prevalence of patients on antibiotics (ABX)

Description by hospital type	ADM	No. of patients on ABX (%)	Adults						Neonates				Paediatrics					
			Medical		Surgical		ICU		Medical		Intensive care		Medical		Surgical		ICU	
			ADM	ABX (%)	ADM	ABX (%)	ADM	ABX (%)	ADM	ABX (%)	ADM	ABX (%)	ADM	ABX (%)	ADM	ABX (%)	ADM	ABX (%)
Primary																		
Hospital 1	122	79 (64.8)	45	30 (66.7)	51	29 (56.9)	0		0		3	3 (100.0)	20	15 (75.0)	3	2 (66.7)	0	
Secondary																		
Hospital 2	150	86 (57.4)	60	26 (43.3)	55	43 (78.2)	0		0		0		13	7 (53.8)	0		22	10 (45.5)
Hospital 3	318	159 (50.0)	127	72 (56.7)	123	44 (35.8)	1	1 (100.0)	0		30	15 (50.0)	37	27 (73.0)	0		0	
* Total*	468	245 (52.4)	187	98 (52.4)	178	87 (48.9)	1	1 (100.0)	0	0	30	15 (50.0)	50	34 (68.0)	0	0	22	10 (45.5)
Tertiary																		
Hospital 4	629	367 (58.4)	158	84 (53.2)	273	145 (53.1)	16	13 (81.3)	65	34 (52.3)	21	14 (66.7)	69	60 (87.0)	25	16 (64.0)	2	1 (50.0)
Hospital 5	988	515 (52.1)	459	238 (51.9)	324	158 (48.8)	7	7 (100.0)	0		86	41 (47.7)	80	55 (68.8)	32	16 (50.0)	0	
Hospital 6	140	93 (66.4)	54	28 (51.9)	58	41 (70.7)	2	2 (100.0)	5	5 (100.0)	5	4 (80.0)	16	13 (81.3)	0		0	
Hospital 7	550	263 (47.8)	122	48 (39.3)	333	150 (45.0)	2	2 (100.0)	52	38 (73.1)	0		41	25 (61.0)	0		0	
* Total*	2307	1238 (53.7)	793	398 (50.2)	988	494 (50.0)	27	24 (88.9)	122	77 (63.1)	112	59 (52.7)	206	153 (74.3)	57	32 (56.1)	2	1 (50.0)
Overall	2897	1562 (53.9)	1025	526 (51.3)	1217	610 (50.1)	28	25 (89.3)	122	77 (63.1)	145	77 (53.1)	276	202 (73.2)	60	34 (56.7)	24	11 (45.8)

ADM, number of patients on admission.

### Drug utilization at 100% (DU100%)

**Figure 2.**
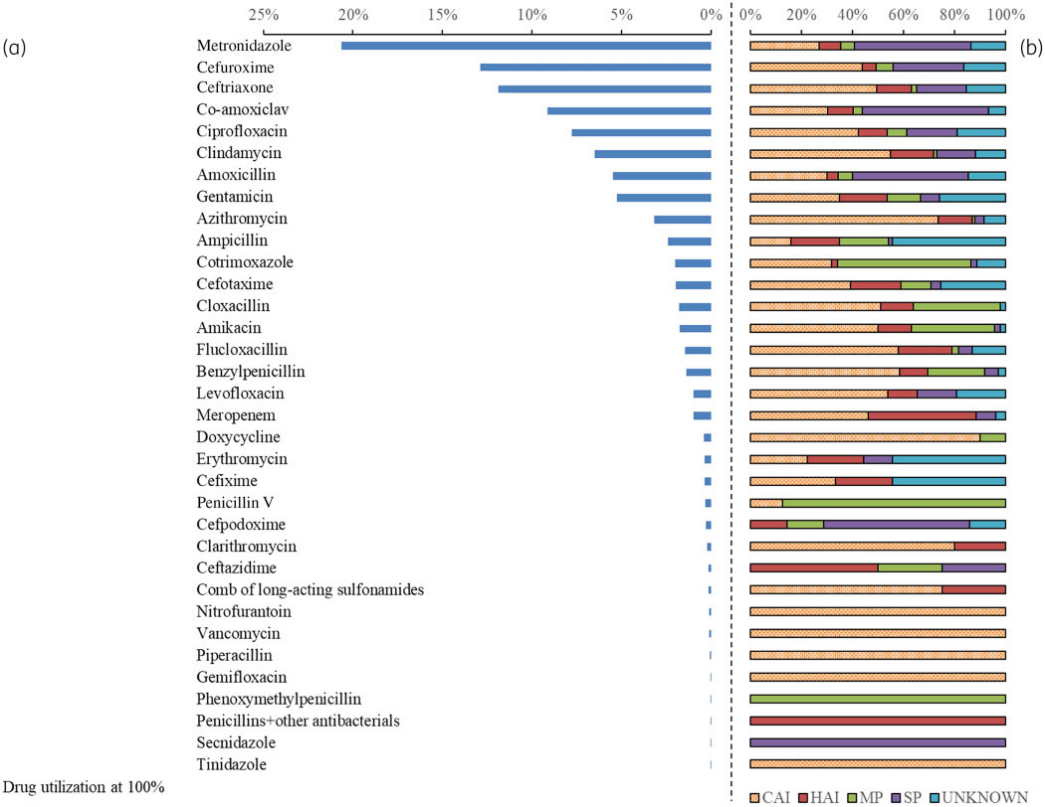
(a) Drug utilization at 100% (DU100%) by ATC level 5 and (b) the indications for use. Abbreviations: CAI, community-acquired infections, HAI, healthcare-associated infections, MP, medical prophylaxis, SP, Surgical prophylaxis.

The majority of antibiotic prescriptions were used for treatment of infections (52.2%, *n = *1367/2620), prophylaxis accounted for 34.1% (*n = *893), and 13.7% (*n = *360) of the prescribed antibiotics had no documented indications. Figure 2 describes DU100% by ATC level 5 and the indications for use. There were 35 different antibiotics prescribed across all hospitals. The five most commonly prescribed antibiotics were metronidazole (*n = *20.6%, *n = *541/2620), cefuroxime (12.9%, *n = *338/2620), ceftriaxone (11.8%, *n = *310/2620), amoxicillin/clavulanic acid (8.8%, *n = *231/2620), and ciprofloxacin (7.8%, *n = *204/2620). This pattern of antibiotic use varied among different age groups. Among neonates, gentamicin (21.6%, *n = *65/301), ampicillin (19.3%, *n = *58/301), cloxacillin (13.3%, *n = *40/301), cefotaxime (12.3%, *n = *37/301) and amikacin (10.6%, *n = *32/301) were the most common antibiotics used (Table S1, available as Supplementary data at *JAC-AMR* Online). Among the adult population, metronidazole (27.1%, *n = *412/1521), cefuroxime (12.7%, *n = *193/1521), ceftriaxone (12%, *n = *183/1521), amoxicillin/clavulanic acid (11.6%, *n = *176/1521) and ciprofloxacin (8.2%, *n = *124/1521) were the top five antibiotics used.

### Antibiotic prescription by type of infection

The majority of antibiotics prescribed for treatment of infections were for community-acquired infections (76.4%%, 1045/1367) and hospital-associated infections (23.6%, 322/1367) (Figure 2). The most common antibiotics prescribed by the anatomic site of infection are shown in Table 3. The top three infections for which antibiotics were prescribed were pneumonia or lower respiratory infections (12.4%, *n = *325/2620), skin and soft tissue (11.0%, *n = *289) and sepsis (8.5%, *n = *223), respectively. For respiratory tract infections, the top three antibiotics used were ceftriaxone (*n = *19.9%, *n = *66/331), azithromycin (18.7%, *n = *62/331) and amoxicillin/clavulanic acid (13.6%, *n = *45/331). The top three antibiotics for treating skin, soft tissue, bone and joint infections were clindamycin (28.8%, *n = *97/337), metronidazole (15.1%, *n = *51/337) and ciprofloxacin (*n = *13.4%, *n = *45/337). The top three antibiotics for treating bloodstream infections and pyrexia of unknown origin were ceftriaxone (11.5%, *n = *39/338), gentamicin (8.0%, *n = *27/338) and cloxacillin (7.1%, *n = *24/338).

**Table 3.** Top five antibiotics (ATC level 5) prescribed for patients by type of infection (September to December 2019)

Reasons for antibiotic use			Top 5 antibiotics used (ATC level 5) among all patients														
	Total		First			Second			Third			Fourth			Fifth		
	no.	(%)	Antibiotic	no.	(%)	Antibiotic	no.	(%)	Antibiotic	no.	(%)	Antibiotic	no.	(%)	Antibiotic	no.	(%)
Infections	1367	(52.2)															
Skin and soft tissue	289	(11.0)	CLI	80	(27.7)	MTZ	51	(17.6)	CIP	38	(13.1)	CXM	37	(12.8)	CTO	20	(6.9)
Bone/joint	48	(1.8)	CLI	17	(35.4)	CXM	9	(18.8)	CIP	7	(14.6)	AMC	5	(10.4)	FLX	2	(4.2)
Lower UTI	90	(3.4)	CXM	52	(57.8)	CIP	12	(13.3)	CTO	9	(10.0)	MTZ	4	(4.4)	AMX	3	(3.3)
Upper UTI	40	(1.5)	CXM	12	(30.0)	CTO	5	(12.5)	CIP	4	(10.0)	GEN, MEM	3	(7.5)	MTZ, NIT, CXM	2	(5.0)
Pneumonia or LRTI	325	(12.4)	CTO	64	(19.7)	AZM	62	(19.1)	AMC	45	(13.8)	MTZ	28	(8.6)	CXM	26	(8.0)
Upper RTI	6	(0.2)	CTO	2	(33.3)	CXM, AMC, GEM, AMX, CTO	1	(16.7)									
Obs/Gynae	60	(2.3)	MTZ	18	(30.0)	AMC	9	(15.0)	CLI	8	(13.3)	GEN	6	(10.0)	CTO	5	(8.3)
GU infections	6	(0.2)	PEN, CXM, DOX GEN, MTZ	1	(16.7)									(0.0)			(0.0)
Sepsis	223	(8.5)	CTO	35	(15.7)	GEN	25	(11.2)	CLX	24	(10.8)	CTX	20	(9.0)	AMK	16	(7.2)
PUO	15	(0.6)	CTO, CIP	4	(26.7)	GEN	2	(13.3)	AZM, CXM, AMC, CLI, LVX	1	(6.7)			(0.0)			(0.0)
CNS	85	(3.2)	CTO	28	(32.9)	MTZ	10	(11.8)	COT	7	(8.2)	CXM, CIP	5	(5.9)	COT, GEN	4	(4.7)
Intra-abdominal	52	(2.0)	MTZ	21	(40.4)	CIP	9	(17.3)	CTO	8	(15.4)	CXM	4	(7.7)	AMC	3	(5.8)
GI	101	(3.9)	MTZ	44	(43.6)	CIP	22	(21.8)	CTO	8	(7.9)	AMX	7	(6.9)	CLR	4	(4.0)
ENT	17	(0.6)	CTO, AMC, MTZ	3	(17.6)	PEN, AMX, GEN	2	(11.8)	CLI, AMK	1	(5.9)						
CVS	3	(0.1)	PEN, AMX, CTO	1	(33.3)												
Other infections	7	(0.3)	AMX, CTO, MTZ, ERY, FLX, GEN, MTZ	1	(14.3)												
Prophylaxis	893	(34.1)	MTZ	276	(30.9)	AMC	121	(13.5)	CXM	116	(13.0)	AMX	73	(8.2)	CTO	57	(6.4)
Medical	209	(8.0)	MTZ	29	(13.9)	AMC, CXM	23	(11.0)	GEN	18	(8.6)	CIP	16	(7.7)	AMK	15	(7.2)
Surgical	684	(26.1)	MTZ	247	(36.1)	AMC	113	(16.5)	CXM	93	(13.6)	AMX	65	(9.5)	CTO	60	(8.8)
Unknown reasons	360	(13.7)	MTZ	65	(18.1)	CXM, CTO	48	(13.3)	GEN	34	(9.4)	AMP	28	(7.8)	CIP	27	(7.5)
All prescriptions	2620		MTZ	541	(20.6)	CXM	338	(12.9)	CTO	310	(11.8)	AMC	231	(8.8)	CIP	204	(7.8)

ATC, Anatomic therapeutic classification; UTI, urinary tract infections; LRTI, lower respiratory tract infections; Obs/Gynae obstetrics and gynaecological infections; GU, genitourinary infections in men; PUO, pyrexia of unknown origin; GI, gastrointestinal tract; ENT, ear, nose and throat; CVS, cardiovascular system; CLI, clindamycin, CXM, cefuroxime, CTO, ceftriaxone; MTZ, metronidazole; PEN, benzylpenicillin; DOX, doxycycline; GEN, gentamicin; CIP, ciprofloxacin; AMC, amoxicillin/clavulanic acid; AMX, amoxicillin; ERY, erythromycin; FLX, flucloxacillin; GEM, gemifloxacin; AZM, azithromycin; LVX, levofloxacin; COT, cotrimoxazole; AMK, amikacin; CTX, cefotaxime; AMP, ampicillin; NIT, nitrofurantoin; CLR, clarithromycin.

### Antibiotic prescriptions for prophylaxis

**Figure 3.**
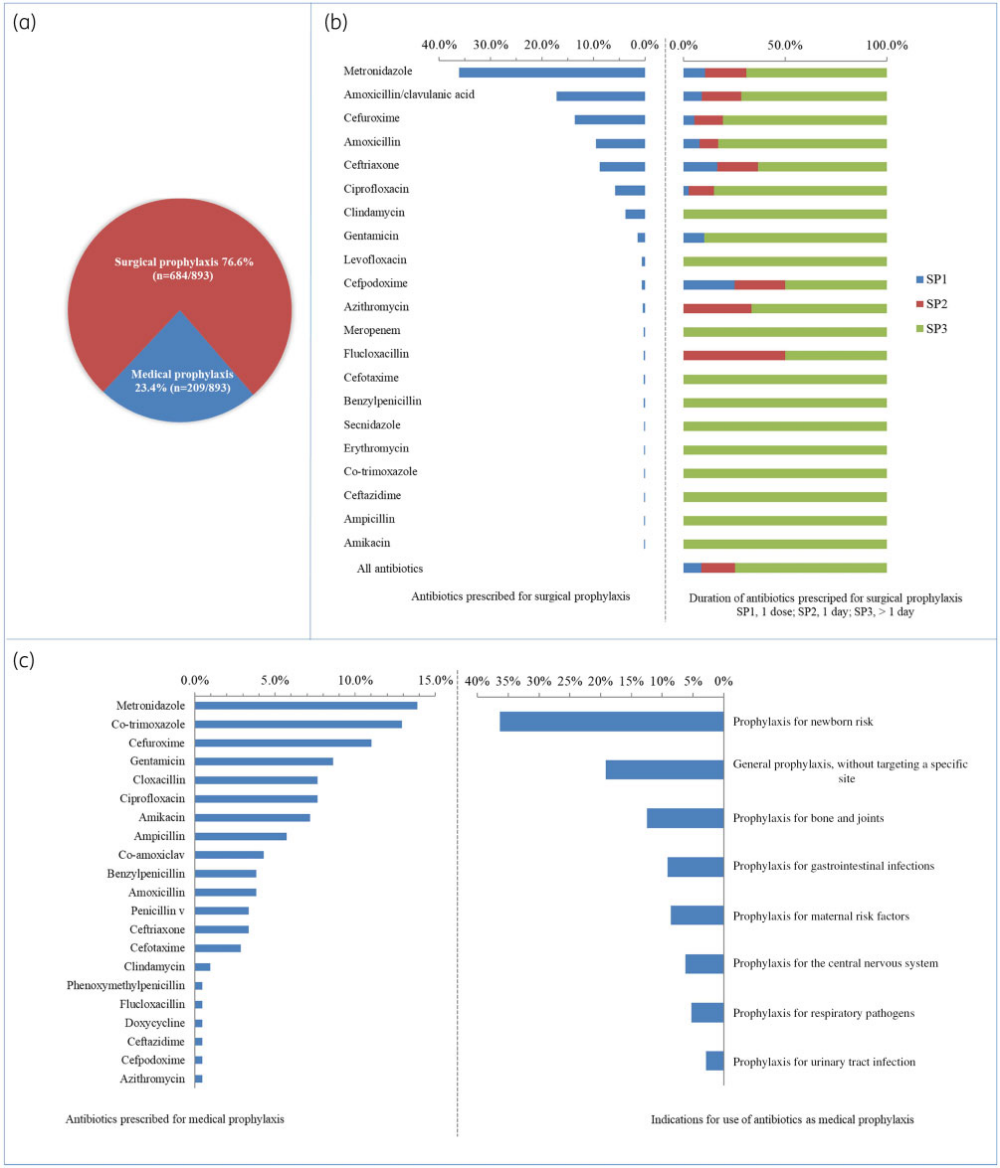
Summary of (a) prophylactic antibiotic use, (b) antibiotics prescribed for surgical prophylaxis and (c) the duration of prescription antibiotics prescribed for medical prophylaxis and the indications for prescription.

Figure 3 shows the distribution of antibiotics for prophylactic use. Prophylaxis was the reason for antibiotic use in 34.1% (*n = *893) of cases. The majority (76.6%, *n = *684/893) were for surgical prophylaxis as compared with medical prophylaxis (23.4%, *n = *209/893). For all patients who received surgical prophylaxis, 74.7% (*n = *511/684) were given for more than 1 day whilst 16.5% (*n = *113) received antibiotics for a day and 8.7% (*n = *60) had received a single dose. Antibiotics prescribed for medical prophylaxis were mainly for managing newborn risk factors such as very low birth weight and intrauterine growth restrictions (36.4%, *n = *76/209). Metronidazole was prescribed more for surgical prophylaxis (36%) than for medical prophylaxis (14%), whilst cotrimoxazole was prescribed mainly for medical prophylaxis (13%).

### Overview of quality of antibiotic prescriptions

Table 4 provides an overview of the quality of antibiotic prescriptions. Of the 1367 antibiotic prescriptions for treatment of infections, approximately 96% (*n = *1314) were empirically prescribed. Thirty-eight of the 53 guided antibiotic treatments targeted at least one multidrug-resistant organism. About 5.2% (*n = *137) of the antibiotic prescriptions were based on biomarkers, largely full blood counts with elevated white blood cell counts (*n = *124, 90.5%). Overall, 48.2% (*n = *1262) of the antibiotic prescriptions had a documented reason in the notes and 46.7% (*n = *1226) had a documented stop/review date indicated.

**Table 4.** Overview of quality of prescription for antimicrobial drugs

Patients	Anti-infective prescriptions	Antibiotic prescriptions, *n* (%)								Antibiotic use guidelines available
		Number	Based on biomarker^a^				Reason in notes	Stop/ review date	Targeted treatment^a^	
			CRP	PCT	WBC	All				
Hospital 1 (*n = *318)	295	273 (92.5)	0	0	4 (2.5)	4 (2.5)	165 (60.4)	49 (17.9)	1 (0.4)	none
Hospital 2 (*n = *150)	154	140 (90.1)	0	0	24 (15.4)	24 (15.4)	47 (33.6)	79 (54.6)	0	none
Hospital 3 (*n = *122)	148	137 (92.5)	0	0	0	0	82 (59.9)	103 (75.2)	0	none
Hospital 4 (*n = *140)	193	161 (83.4)	0	0	13 (82.9)	13 (82.9)	108 (67.1)	113 (70.2)	3 (1.9)	none
Hospital 5 (*n = *629)	700	609 (87.0)	0	0	37 (23.7)	37 (23.7)	314 (51.6)	406 (66.7)	10 (1.6)	none
Hospital 6 (*n = *988)	967	890 (92.3)	7 (0.8)	6 (0.7)	34 (21.8)	46 (27.2)	388 (43.6)	201 (22.6)	31 (3.5)	none
Hospital 7 (*n = *550)	418	410 (98.1)	0	0	44 (28.2)	44 (26.0)	158 (38.5)	275 (67.1)	8 (2.0)	none
Total (*n = *2897)	2875	2620 (91.1)	7 (0.5)	6 (0.4)	156 (11.4)	169 (12.4)	1262 (48.2)	1226 (46.7)	53 (3.9)	–

a Antibiotic prescriptions for systemic therapeutic use only (i.e., healthcare-associated or community-acquired infection, *n = *1367); CRP, C-reactive protein; PCT, procalcitonin; WBC, white blood cells.

## Discussion

In this multicentre study of about 3000 hospitalized patients, one in every two hospitalized patients received at least one antibiotic and over 60% of the prescriptions included ≥2 drugs for a single indication. Approximately half of the antibiotics were used for managing infections, one-third for prophylaxis and about a tenth had no documented indication.

Prevalence rates of antibiotic use in this study are similar to previously published studies from Ghana. In a multicentre PPS conducted in 10 hospitals across Ghana, nearly 61% of patients surveyed were on antibiotics,^14^ with 71% prevalence of antibiotic use in paediatric and surgical patients.^8^^,^^15^ Other studies have shown a prevalence of 51.4%, 57.1% and 55.6% for antimicrobial use in three different hospitals in Ghana.^7^^,^^16^ Findings from our study are similar to antibiotic use data reported from many African institutions,^17^^,^^18^ but higher compared with reports in many reviews spanning several other regions.^19^^,^^20^ We realized that the context in which antibiotics are prescribed in sub-Saharan Africa is similar and there is a consensus that antibiotic use in the sub-region is high.^17^ Such high antibiotic prescription rates in hospitals are fuelled by factors such as inadequate diagnostic microbiology services and differences in the organizational structures of hospitals.^21^ Given the association between antimicrobial use and the selection of resistant pathogens, the high frequency of antimicrobial use in Ghana is a reflection of the AMR problem in the country.^22^ The problem is further compounded by the observation that physicians may not even be aware of the AMR threats associated with antibiotic use.^23^

Point prevalence surveys of antibiotic use, when repeated regularly, provide data on patterns of antibiotic use and serve as a benchmark for antibiotic stewardship activities. Observations from previous multicentre PPS in Ghana,^14^^,^^16^^,^^24^ including findings from this current study, suggest four major antibiotic prescribing indicators for antimicrobial stewardship (AMS) interventions. First, metronidazole, ceftriaxone, cefuroxime, and amoxicillin/clavulanic acid constitute good candidates for AMS because of their high frequency of prescription in Ghana. The frequent use of these antibiotics across all hospitals suggests that at least a proportion of their prescribing could be inappropriate.^19^ Second, the majority of antibiotics are prescribed empirically without supporting microbiological data, even in facilities where microbiological services were available. This situation reflects the low utilization of diagnostic microbiology services in Ghana and other low-resource settings.^14^^,^^21^^,^^25^ Correct infection diagnosis and antibiotic treatment require the existence of clinical microbiology services and the involvement of diagnostic stewardship.^19^^,^^26^ Improved access to diagnostic microbiology services is a recognized key metric for AMS interventions in LMICs, with known advantages of reducing inappropriate antibiotic use and healthcare cost.^27^ Efforts are needed to improve microbiological culture utilization before antibiotic prescribing. Such efforts may include improved financing of cultures through health insurance schemes as well as education of healthcare providers on the need to perform cultures. Third, there is a high proportion of prophylactic antibiotic use for a range of indications, but this is unusually high for surgical prophylaxis lasting >1 day. The side effects of prolonged prophylaxis are well documented in the literature, with an increased risk of AMR development.^19^^,^^28^^,^^29^ Last, documentation of the reason for antibiotic prescription and stop/review dates are uncommon and represent opportunities for AMS. Such documentation facilitates appropriate communication of diagnosis and treatment among healthcare staff and allows an informed de-escalation of drugs.^2^^,^^17^^,^^19^

In the last 15 years, there has been a steady trickle of papers on AMR in Ghana.^22^^,^^30–32^ Many of these papers end with a call for improved AMR surveillance and implementation of AMS activities. Ghana’s national AMR landscape is young and lacks AMS intervention programmes. In 2018, Ghana launched its AMR Policy and the National Action Plan.^33^^,^^34^ Two of the Policy’s five core objectives seek to address AMS via optimized use of antimicrobial agents, improve awareness and understanding of antimicrobial use through effective communication, education and training. Our study aligns with the national strategy and highlights the need to institute AMS programmes ‒ within the context that the prevalence of antibiotic use is high ‒ to promote behaviour change in antibiotic prescribing practices. It is important to note that most hospitals in Ghana do not possess hospital-specific guidelines for antimicrobial use. The Standard Treatment Guidelines of Ghana outline preferred treatments for common health problems including infections but do not provide specific guidelines tailored according to the individual needs of institutions.^35^ Local antibiotic guidelines improve the optimal use of antibiotics, promote behaviour change in antibiotic prescribing and dispensing practices, and build the best-practices capacity of healthcare professionals regarding the rational use of antibiotics.^26^ The WHO provides a practical toolkit for AMS in healthcare facilities in LMICs.^26^ The toolkit advocates institutional antimicrobial guidelines informed by available resources, local antibiogram, and benchmarking standards for quality indicators of antibiotic prescribing.

Our study has some limitations. Although we noted similarities in the prevalence of antibiotic prescribing within hospitals and between regions, as well as between previously reported PPS, the data may not be representative for most of the country. For instance, private healthcare facilities were not surveyed, and there was low representation from primary hospitals, and the overall rates provided are averages. Our analysis did not control for institutional factors, which may influence antibiotic prescribing patterns. There is the possibility that the quality of data may have been affected by poor record-keeping, leading to possible underestimation of antimicrobial use.

### Conclusions

On average, one in every two patients in our study received an antibiotic, with relatively high use of metronidazole and cephalosporins, especially for community-acquired infections. Second, the majority of antibiotics were prescribed empirically without culture. There is an urgent need to improve access to bacterial culture and susceptibility testing to inform antibiotic prescribing. Third, documentation of the reason for antibiotic prescription and stop/review dates were uncommon. Findings from this study could be used as benchmarks for quality improvement of antibiotic prescribing. These indicators should be targeted as key interventions, and the effects of such interventions should be measured with repeated point prevalence surveys.

## Supplementary Material

dlab087_Supplementary_Data
