# Supplementary material for: Antimicrobial use in hospitalized patients: a multicentre point prevalence survey across seven hospitals in Ghana
Source: JAC Antimicrob Resist. 2021 Jul 12;3(3):dlab087. doi: 10.1093/jacamr/dlab087 (PMC8275021; doi:10.1093/jacamr/dlab087)
Supplement: dlab087_Supplementary_Data [file dlab087_supplementary_data.docx]

**Supplementary data**

Table S1: Top five antibiotics (ATC level 5) prescribed according to age groups

|  |  |  | Top five antibiotics prescribed | | | | | | | | | | | | | | | | | | | |
| --- | --- | --- | --- | --- | --- | --- | --- | --- | --- | --- | --- | --- | --- | --- | --- | --- | --- | --- | --- | --- | --- | --- |
|  |  |  | 1 | | |  | 2 | | |  | 3 | | |  | 4 | | |  | 5 | | |  |
| Age groups | Total |  |  | no | (%) |  |  | no | (%) |  |  | no | (%) |  |  | no | (%) |  |  | no | (%) |  |
| Neonates | 301 |  | GN | 65 | (21.6) |  | AMP | 58 | (19.3) |  | CLX | 40 | (13.3) |  | CTX | 37 | (12.2) |  | AMK | 32 | (10.6) |  |
| Infants | 128 |  | CTR | 19 | (14.8) |  | CXM,GN | 17 | (13.3) |  | CTX | 14 | (10.9) |  | MTZ | 13 | (10.2) |  | PEN | 9 | (7.0) |  |
| Paediatrics | 334 |  | CTR | 61 | (18.3) |  | MTZ | 48 | (14.4) |  | CXM | 45 | (13.5) |  | CIP | 34 | (10.2) |  | GEN | 24 | (7.2) |  |
| Children | 124 |  | MTZ | 33 | (26.6) |  | CTR | 23 | (18.5) |  | CXM | 21 | (16.9) |  | AMZ | 11 | (8.9) |  | CLI, CIP | 11 | (8.9) |  |
| Adults | 1521 |  | MTZ | 412 | (27.1) |  | CXM | 193 | (12.7) |  | CTR | 183 | (12.0) |  | AMC | 176 | (11.6) |  | CIP | 124 | (8.2) |  |
| Elderly | 212 |  | CXM | 35 | (16.5) |  | CLI | 28 | (13.2) |  | MTZ | 26 | (12.3) |  | CIP, CTR | 24 | (11.3) |  | AZT | 15 | (7.1) |  |
| Overall | 2620 |  |  |  |  |  |  |  |  |  |  |  |  |  |  |  |  |  |  |  |  |  |

*** CLI, clindamycin, CXM, cefuroxime, CTR, ceftriaxone; MTZ, metronidazole; PEN, benzylpenicillin; DOX, doxycycline; GN, gentamicin; CIP, ciprofloxacin; AMC, amoxicillin/clavulanic acid; AMX, amoxicillin; ERY, erythromycin; FLX, flucloxacillin; GEM, gemifloxacin; AZM, azithromycin; LEV, levofloxacin; COT, cotrimoxazole; AMK, amikacin; CTX,cefotaxime; AMP, ampicillin; NIT, nitrofurantoin; CLR, clarithromycin.
